# Altered expression of β-galactosidase-1-like protein 3 (Glb1l3) in the *retinal pigment epithelium (RPE)-specific 65-kDa protein knock-out* mouse model of Leber’s congenital amaurosis

**Published:** 2011-05-07

**Authors:** Joane Le Carré, Daniel F. Schorderet, Sandra Cottet

**Affiliations:** 1 IRO, Institute for Research in Ophthalmology, Sion, Switzerland; 2 Department of Ophthalmology, University of Lausanne; Switzerland; 3 School of Life Sciences, Federal Institute of Technology (EPFL), Lausanne, Switzerland

## Abstract

**Purpose:**

In this study, we investigated the expression of the gene encoding β-galactosidase (Glb)-1-like protein 3 (Glb1l3), a member of the glycosyl hydrolase 35 family, during retinal degeneration in the retinal pigment epithelium (RPE)-specific 65-kDa protein knockout (*Rpe65^−/−^*) mouse model of Leber congenital amaurosis (LCA). Additionally, we assessed the expression of the other members of this protein family, including β-galactosidase-1 (Glb1), β-galactosidase-1-like (Glb1l), and β-galactosidase-1-like protein 2 (Glb1l2).

**Methods:**

The structural features of Glb1l3 were assessed using bioinformatic tools. mRNA expression of Glb-related genes was investigated by oligonucleotide microarray, real-time PCR, and reverse transcription (RT) -PCR. The localized expression of Glb1l3 was assessed by combined in situ hybridization and immunohistochemistry.

**Results:**

Glb1l3 was the only Glb-related member strongly downregulated in *Rpe65^−/−^* retinas before the onset and during progression of the disease. *Glb1l3* mRNA was only expressed in the retinal layers and the RPE/choroid. The other Glb-related genes were ubiquitously expressed in different ocular tissues, including the cornea and lens. In the healthy retina, expression of *Glb1l3* was strongly induced during postnatal retinal development; age-related increased expression persisted during adulthood and aging.

**Conclusions:**

These data highlight early-onset downregulation of *Glb1l3* in *Rpe65*-related disease. They further indicate that impaired expression of *Glb1l3* is mostly due to the absence of the chromophore 11-*cis* retinal, suggesting that *Rpe65* deficiency may have many metabolic consequences in the underlying neuroretina.

## Introduction

Leber congenital amaurosis (LCA) is the earliest and most severe form of inherited retinal dystrophies, characterized by blindness or severe visual impairment from birth. This disease is generally inherited in an autosomal recessive manner. Mutations have been identified, among others, in the retinal pigment epithelium (RPE)-specific 65-kDa protein (RPE65) [1]. *RPE65* encodes an abundant and evolutionarily conserved 533-amino acid protein, identified as the isomerase of the visual cycle responsible for the synthesis of the chromophore 11-*cis*-retinal [2-6]. It is currently estimated that *RPE65* mutations account for approximately 10% of all severe, childhood-onset retinal dystrophies [7]. Patients affected by these mutations show visual defects, including marked RPE damage and a strongly reduced, or absent, electroretinogram [8,9]. A common fate in retinal dystrophies is the loss of photoreceptors, yet the relationship between genetic mutation, retinal defect, and final photoreceptor apoptosis remains largely unresolved.

*Rpe65^−/−^* mice are characterized by a massive accumulation of all-*trans*-retinyl esters in droplets and an absence of 11-*cis*-retinoids in retinal tissues, consistent with the role of Rpe65 as a retinoid isomerase [2]. Woodruff and colleagues [10] showed that, in these mice, the constitutive activity of unliganded opsin is sufficient to trigger photoreceptor apoptosis.

In a previous gene expression-profiling study of *Rpe65^−/−^* mice, we reported that 130 known genes were downregulated during disease progression [11]. The observation that, besides these known genes, some of unknown functions also decreased in diseased mice prompted us to further investigate β- galactosidase (Glb)-1-like protein 3 (Glb1l3) in the retina. Glb1l3 is a member of the glycosyl hydrolase 35 family of proteins (EC 3.2.1.23) [12]. These proteins display hydrolase activity catalyzing the cleavage of lactose, as well as galactosyl residues from gangliosides, glycoproteins, and glycosaminoglycans [13]. However, the function of these proteins in retinal tissue is largely unknown.

## Methods

### Mouse lines and genotyping

These studies adhered to the Association for Research in Vision and Ophthalmology (ARVO) statement for the use of animals in ophthalmic and vision research, and were approved by the veterinary service of the State of Valais (Switzerland). Wild-type (WT) C57BL/6 mice were purchased from Charles River Laboratories (Les Oncins, France). *Rpe65^−/−^* mice had a C57BL/6 genetic background (from Dr. T.M. Redmond, National Institutes of Health, Bethesda, MD) [2]. The genotype of the mice was determined by PCR analysis with genomic DNA isolated from tail tissue, as described in [2]. Briefly, genomic DNA was used to screen for the presence of Rpe65 by PCR as follows: denaturation at 95 °C for 5 min, followed by 35 cycles of 95 °C for 1 min, 61 °C for 1 min and 72 °C for 1 min. We used triple primer sets to discriminate either the *Rpe65* null gene from the wt gene. Animals were kept in a 12 h:12 h light-dark cycle with unlimited access to food and water.

### Tissue isolation and RNA extraction

Age-matched animals were killed by cervical dislocation. Retinal tissues from each mouse were quickly isolated in RNA*later* (Ambion, Huntingdon, UK) before being transferred to TRIzol (Invitrogen, Basel, Switzerland) and stored at −80 °C until RNA extraction. Total RNA was extracted according to manufacturer’s instructions, and its amount was determined by Ribogreen assay (Invitrogen).

### Oligonucleotide microarray

Oligonucleotide microarray analysis was done as elsewhere described in detail [11]. Briefly, 1 μg of total RNA was used to generate double-stranded cDNA, which was used as a template for biotinylated cRNA synthesis using an Affymetrix GeneChip Expression 3′-Amplification Kit for IVT Labeling (Affymetrix, Santa Clara, CA). Next, 20 μg of target cRNA were fragmented and hybridized on Affymetrix Mouse Genome 430 2.0 GeneChips. The washed chips were scanned with an Affymetrix GeneChip Scanner 3000 using the GCOS software (Affymetrix). Data normalization was performed using the robust multi-array analysis (RMA) algorithm as implemented in the GeneSpring 7.2 software (Agilent Technologies, Waldbronn, Germany). Triplicate analyses were performed for each condition studied.

### Reverse transcription (RT)–PCR analysis

One microgram of total RNA in a 30 μl reaction was used for cDNA synthesis using oligo (dT)_18_ according to the manufacturer’s procedure (AffinityScript Reverse Transcriptase; Agilent, Basel, Switzerland). The equivalent of 20–100 ng of original total RNA was used for PCR using 2× Master Mix (Qiagen, Basel, Switzerland) and 1 μM forward and reverse primer pairs. PCR was performed with the following cycling conditions: 35 cycles of denaturation at 95 °C for 1 min, annealing at 60 °C for 30 s, and extension at 72 °C for 30 s. Primers (Eurogentec, Seraing, Belgium; Table 1) were designed to span several exons of the target gene. cDNA amplification of the housekeeping gene encoding the ribosomal protein L8 (*Rl8*) was used as positive control. PCR products were run on 1.5% agarose gel stained with SYBR Safe (Invitrogen).

**Table 1:** Primer sequences for PCR analysis and in situ hybridization.

**Symbol**	**Forward primer (5′-3′)**	**Reverse primer (5′-3′)**	**GenBank accession number**
*Glb1*	GGCGTGTGAACTATGGCAGA	AAGACCGTCCAGTTGGTGAG	NM_009752
*Glb1l*	CGAGCCTATGTGATGGTAGA	TGGTCCATCCAGGTAGATAG	BC021773
*Glb1l2*	CATGGAGAACCTGCCAGTAA	ACATTCCAGTAGCGTCCAAG	BC038479
*Glb1l3*	TACTGGACTGGCTGGTATGA	CAGAGGCAGGTAGAATGAGA	NM_001113323
*Rl8*	ACTGGACAGTTCGTGTACTG	GCTTCACTCGAGTCTTCTTG	NM_012053
*Gapdh*	GAGGCCGGTGCTGAGTATGT	GGTGGCAGTGATGGCATGGA	M32599

### Real-time PCR analysis

The equivalent of 20–50 ng of original total RNA was used for PCR amplification using the 2× brilliant SYBR Green QPCR Master Mix (Agilent) with either 125 nM (glyceraldehyde 3-phosphate dehydrogenase [*GAPDH*], *Glb1l3*), 250 nM (β-galactosidase-1 [*Glb1*]*,* β-galactosidase-1 like [*Glb1l*], β-galactosidase-1-like protein 2 [*Glb1l2*]), or 400 nM (ribosomal protein L8 [*Rl8*]) forward and reverse primer pairs. Real-time PCR was performed in triplicate in a Mx3000P system (Agilent) with the following cycling conditions: 40 cycles of denaturation at 95 °C for 30 s, annealing at 55 °C for 30 s, and extension at 72 °C for 60 s. Semiquantitative values were obtained by the cycle number (cycle threshold [C_t_] value) reflecting the point at which fluorescence started to increase above background at a fixed threshold level. Values obtained for the target genes were normalized with the housekeeping genes *GAPDH* or *Rl8*. For primer sequences, see Table 1.

### In situ hybridization

Murine eyes were enucleated and fixed for 30 min in ice-cold 4% paraformaldehyde (PFA)/1×-phosphate buffer saline (PBS; 154 mM NaCl, 1 mM KH_2_PO_4_, 3 mM Na_2_HPO_4_ heptahydrate-diethylpyrocarbonate [DEPC]) and transferred to a 30% sucrose/H_2_O-DEPC solution overnight at 4 °C. Fixed and cryopreserved eyes were embedded in Yazzulla (30% albumin/3% gelatine in PBS-DEPC) and cut into 10 μm-thick cryosections using a Leica CM1900 cryostat (Leica Microsystems, Heerbrugg, Switzerland). Eye sections were thawn-mounted on Vectabond Reagent-treated (Vector Laboratories, Burlingame, CA) SuperFrost Plus glass slides (Menzel Gläser, Braunschweig, Germany). PCR-amplified *Glb1l3* cDNA fragments were subcloned into pGEM-T Easy Vector (Promega, Madison, WI) according to the manufacturer’s protocol. Plasmids were linearized with SpeI or ApaI restriction enzymes to obtain a template sequence for either the sense or antisense RNA probes. Digoxygenin (DIG)-labeled antisense and sense cRNA probes were synthesized by in vitro transcription of 1–2 μg of linearized DNA with either T7 or Sp6 RNA polymerases (DIG RNA labeling Mix; Roche, Basel, Switzerland) according to the manufacturer’s protocol. DIG-labeled sense and antisense probes were tested by immunodot blotting and equal amounts of probe (1:100 dilution) used in the experiment. In situ hybridizations were performed in hybridization buffer (4× SSC, 50% formamide, 1× Denhardt’s, 0.2 mg/ml salmon sperm) at 60 °C for 40 h. Slides were washed (2× for 15 min in 2× SSC at room temperature [RT], 30 min in 1× SSC at 60 °C, 30 min in 0.5× SSC at 60 °C, 2× 15 min in 0.5× SSC at RT) and then incubated 2 h at RT with anti-DIG antibodies conjugated with alkaline phosphatase (AP; Roche) diluted to 1:3,000. Color staining was developed by incubating sections with NBT/BCIP (Roche) in the dark at RT. The reaction was stopped by adding TE (1:10), followed by a final wash overnight in 95% EtOH.

### Immunohistochemistry

Slides from in situ hybridization were rehydrated in deionized water for 15 min before immunohistochemistry. In brief, retina sections were blocked for 1 h at RT: in PBS with 2% normal goat serum (NGS; Sigma, Buchs, Switzerland) and 0.2% Triton X-100 (Sigma) for recoverin and calbindin D-28K antibodies; in PBS with 0.2% gelatin and 0.1% Triton X-100 for Brn-3 antibody; in PBS with 3% BSA, 5% NGS, and 0.2% Triton X-100 for cone arrestin antibody; and in PBS with 10% NGS and 0.3% Triton X-100 for glial fibrillary acidic protein (GFAP) antibody. Sections were incubated overnight at 4 °C with primary antibodies in the blocking buffer, except for Brn-3 antibody (in PBS with 2% FBS). Sections were blocked again in blocking buffer for 30 min at RT before being incubated with fluorochrome-conjugated secondary antibody for 1 h at RT. Incubation with secondary antibody alone was used as a negative control. Species and dilutions of the antibodies used were as follows: rabbit anti-recoverin (1:1,000; AB5585; Chemicon International, Temecula, CA); rabbit anti-calbindin D-28K (AB1778; 1:500; Chemicon International); rabbit anti-cone arrestin (1:100; LUMIJ from Dr. C. Craft; Doheny Eye Institute, Los Angeles, CA); goat anti-brn-3 (sc-6026; 1:100; Santa Cruz Biotechnology, Santa Cruz, CA); rabbit anti-GFAP (Z 0334; 1:400; DakoCytomation, Glostrup, Denmark); Alexa Fluor 594 goat anti-rabbit IgG (1:1,000; Invitrogen); and Alexa Fluor 594 donkey anti-goat IgG (1:200; Invitrogen). Following three washes in PBS, sections were mounted in Citifluor AF1 (Citifluor Ltd., London, UK).

### Bioinformatic tools

Amino acid sequence alignment was performed using the multiple sequence alignment program T-Coffee version 5.05 from EMBL-EBI. Editing of multiple alignment results was done using the multiple alignment editor Jalview [14].

### Statistical analysis

Data were analyzed by a two-way ANOVA (ANOVA), using genotype and age factors (GraphPad Prism 5.0; GraphPad Software Inc., La Jolla, CA).

## Results

### Differential expression of Glb-related transcripts in Rpe65^−/−^ retinas

**Figure 1:**
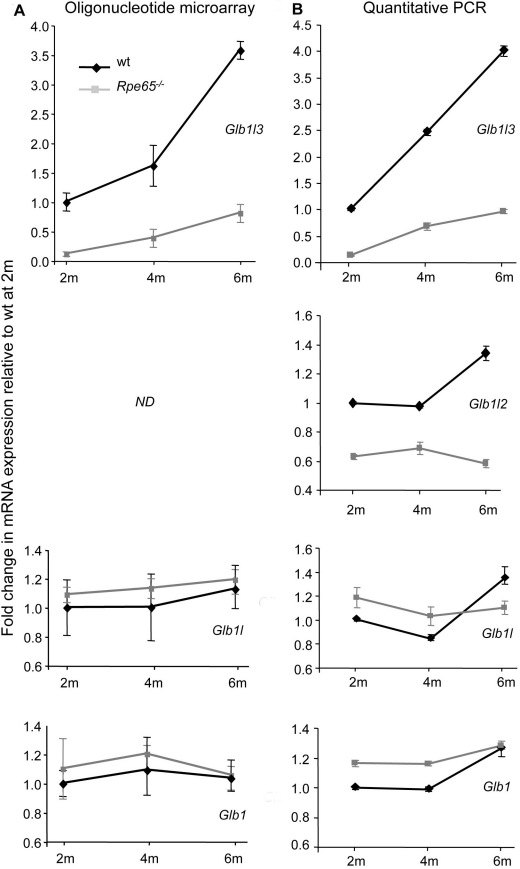
Transcriptional alteration of β-galactosidase (Glb)-related members in *Rpe65^−/−^* retinas. **A**: mRNA expression from wild-type (wt, black diamonds) and *Rpe65^−/−^* retinas (gray squares) was assessed at 2, 4 and 6 months of age (2–6 m) by oligonucleotide microarray. **B**: Retinal mRNA expression was validated by real-time PCR. Relative mRNA levels were expressed as fold inductions relative to samples from 2-month-old WT mice arbitrarily set to 1. Downregulated mRNA expression in diseased retinas at all ages was specific to Glb1l3 (p<0.001 by two-way ANOVA (ANOVA) for *Rpe65^−/−^* versus WT), and to a less extent to Glb1l2 (p<0.05 by two-way ANOVA for *Rpe65^−/−^* versus WT), while expression of the other Glb-related members remained unaltered. Data are the mean±standard error of the mean (SEM) of 3 (**A**) and 4 (**B**) independent animals. *ND*: not described; the *Glb1l2* gene was not present in the Affymetrix Mouse Genome 430.2 Genechip.

In this study, the retinal expression of Glb-related members was investigated in 2-, 4-, and 6-month-old WT and *Rpe65^−/−^* mice by oligonucleotide microarray (Figure 1A) and real-time PCR analysis (Figure 1B). In WT mice, the level of *Glb1l3* transcript was shown to progressively increase with age. However, *Glb1l3* mRNA expression was strongly downregulated at all ages in *Rpe65^−/−^* mice, compared to WT levels (p<0.001). Altered mRNA expression was restricted to *Glb1l3* and *Glb1l2*, while transcripts of the other Glb-related members remained unchanged.

### Features of the amino acid sequences of the mouse Glb-related proteins

**Figure 2:**
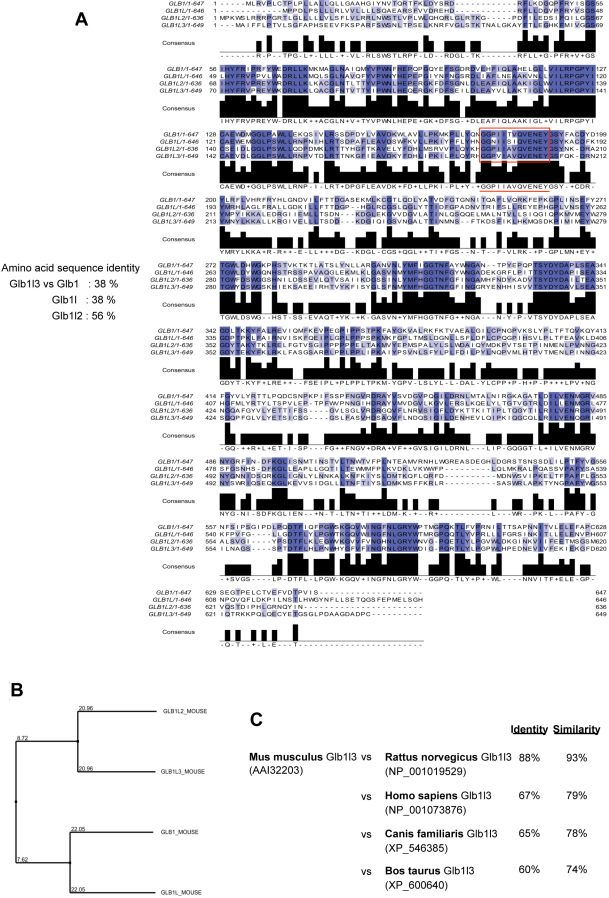
Amino acid conservation between mouse Glb-related proteins. **A**: Multiple alignment of protein sequences derived from the UniProt database showed the degree of conservation between mouse β-galactosidase (Glb)-related members. **B**: Phylogenic tree displayed the degree of evolutionary conservation between the Glb-related proteins. **C**: Pairwise alignment of the mouse sequence against the mammalian homologs retrieved from the NCBI Homologene database.

**Figure 3:**
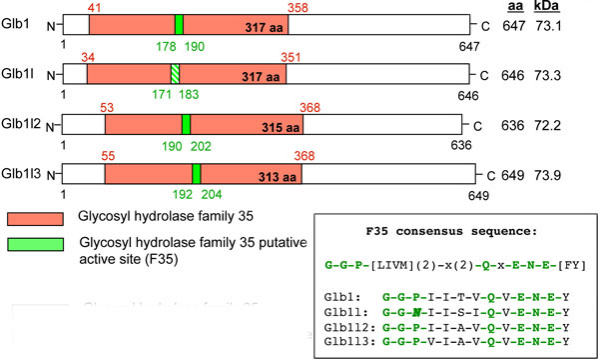
Conserved protein domains in mouse Glb-related proteins. All Glb-related proteins are characterized by the glycosyl hydrolase family 35 domain (red box) and its putative F35 active site (green box). The F35 consensus sequence of Glb1l (hatched green box) differs by one amino acid (N instead of P) from the canonical motif.

Characterization of the Glb-related proteins was further assessed using database searches and computational sequence analyses. The *Glb1l3* gene is localized on chromosome 9, and it contains 21 exons (ENSMUSG00000031966). The *Glb1l3* and *Glb1l2* genes are close to each other in a tail-to-head position at location 9A4. The *Glb1* gene (ENSMUSG00000045594) is located on the same chromosome, telomeric of *Glb1l3* at location 9F3, whereas the *Glb1l* gene is located on chromosome 1. The multiple alignment of Glb-related amino acid residues revealed that Glb1l3 protein presented the highest degree of homology with Glb1l2 (56% of identity) and 38% with both Glb1 and Glb1l proteins (Figure 2A). The phylogenic tree in Figure 2B obtained from amino acid sequence alignment displayed the degree of evolutionary conservation between the Glb-related members, especially the close relationship between Glb1l3 and Glb1l2 proteins. Mammalian homologs showing a high degree of conservation with Glb1l3 mouse protein were assessed using the NCBI Homologene database. Following pairwise alignment of the mouse sequence against the other homologous sequences, the highest degree of identity at the protein level was observed for the rat homolog (88%), followed by the human homolog (67%) (Figure 2C). As depicted in Figure 3, all Glb-related mouse proteins were characterized by the glycosyl hydrolase family 35 domain, as determined in the Pfam database (PF01301). Moreover, Glb1, Glb1l2, and Glb1l3 members possessed a 12-amino acid-long conserved domain (consensus sequence GGPIIAVQVENEY), identified as being the putative F35 active site of the glycosyl hydrolases family 35 (Figure 2A and Figure 3). The consensus pattern of the glycosyl hydrolases family 35 putative active site, as established from the PROSITE database (PROSITE entry PS01182), is the following: G-G-P-[LIVM](2)-x(2)-Q-x-E-N-E-[FY]. The sequences known to belong to this class are all detected by the consensus pattern, although no other sequence showing this pattern was detected in the UniProtKB/Swiss-Prot protein database. In Glb1l sequence, proline, the third amino acid of the putative glycosyl hydrolase family 35 motif active site, is replaced by an asparagine, indicating that at this position, some variation may be acceptable.

### Characterized expression of Glb-related members in the adult mouse eye

**Figure 4:**
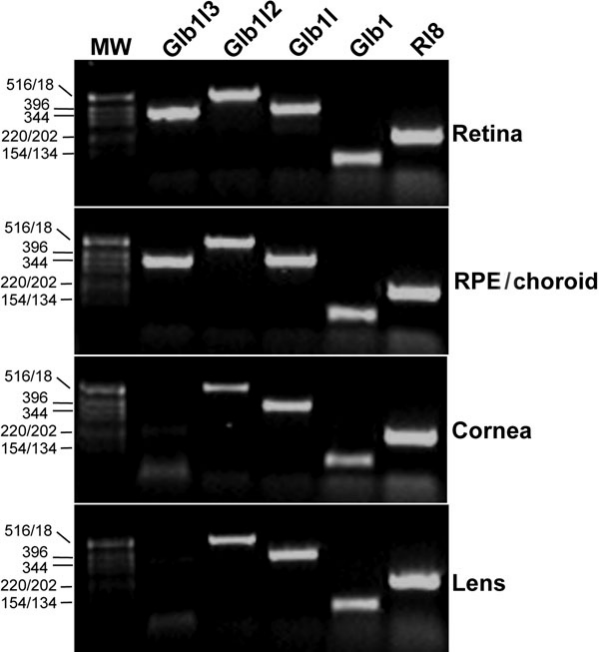
Expression of *Glb*-related genes in the eye. Specific cDNA products for *Glb1* (92 bp), *Glb1l* (368 bp), and *Glb1l2* (523 bp) were ubiquitously expressed in the different ocular tissues, whereas expression of *Glb1l3* transcript (363 bp) was restricted to the retina and the RPE/choroid. *Rl8* mRNA expression (198 bp) was used as an internal standard. Molecular weight (MW), DNA ladder in base pairs (bp).

The localized expression of Glb-related members in the eye has not been previously addressed. We assessed Glb-related mRNA expression in ocular tissues from 2-month-old WT mice by RT–PCR (Figure 4). Expression of Glb1l3 was restricted to the retina and the RPE/choroid, while the other Glb-related members were ubiquitously expressed in the cornea and lens ocular tissues. Detection of ubiquitous Rl8 transcript was used as a control for PCR amplification.

### Expression of *Glb1l3* mRNA in adult wild-type and Rpe65^−/−^ retinas

**Figure 5:**
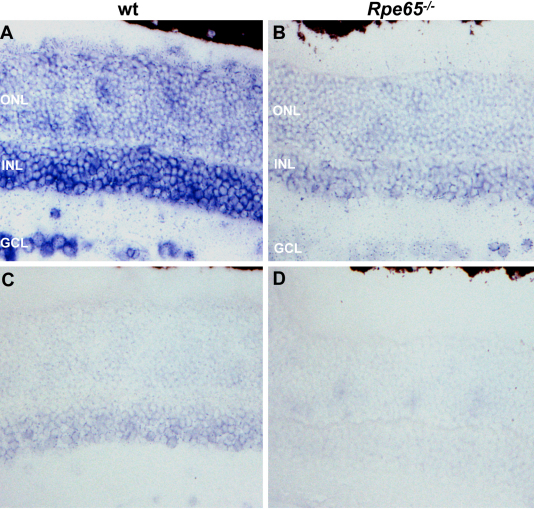
Expression of *Glb1l3* mRNA in 2-month-old wild-type and *Rpe65^−/−^* retinas. **A**: In situ hybridization was performed on retina section from wild-type mice. **B**: In situ hybridization was performed on retina section from *Rpe65^−/−^* mice. **A**, **B**: Wild-type and *Rpe65^−/−^* retina sections were hybridized with the antisense probe, respectively. **C**, **D**: Wild-type and *Rpe65^−/−^* retina sections were hybridized with the control sense probe, respectively.

We assessed *Glb1l3* mRNA expression in 2-month-old WT (Figure 5 A,C) and *Rpe65^−/−^* (Figure 5 B,D) retinas by in situ hybridization with antisense (Figure 5 A,B) and sense (Figure 5 C, D) probes. *Glb1l3* was expressed in the outer nuclear layer (ONL), inner nuclear layer (INL), and ganglion cell layer (GCL) of the healthy (Figure 5A) and diseased (Figure 5B) retinas. In addition, the level of *Glb1l3* mRNA was markedly reduced in *Rpe65^−/−^* retinas, corroborating the observation made by real-time PCR analysis (Figure 1).

**Figure 6:**
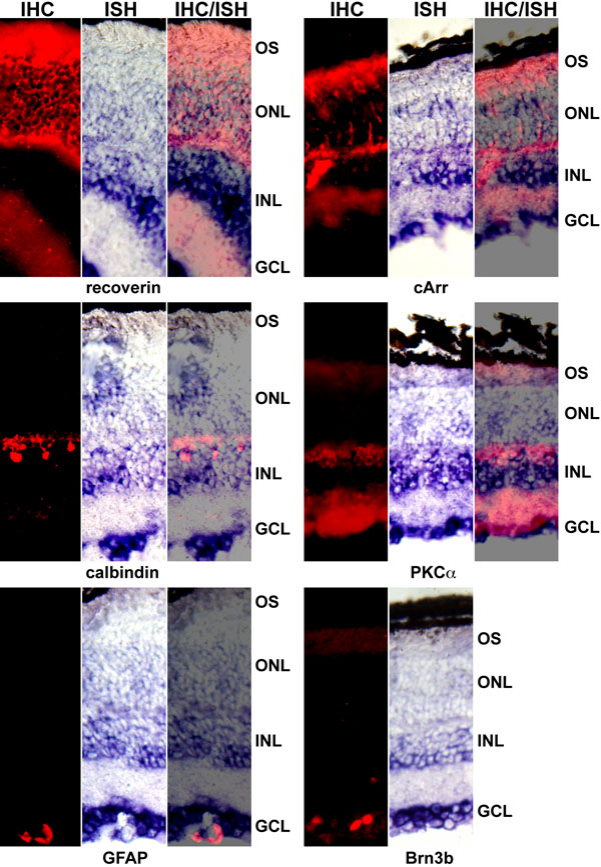
Localized expression of Glb1l3 in retinal cell layers. Wild-type retina sections were used to perform combined in situ hybridization (ISH) and immunohistochemistry (IHC) using cell type-specific retinal markers of the rods (recoverin), the cones (cone arrestin; cArr), the horizontal (calbindin) and bipolar (PKCα) cells of the INL, the Müller cells (GFAP), and the ganglion cells (Brn3b) of the GCL. As the staining for Brn3b was too weak to be detected in a combined experiment, two adjacent retina sections were used for *Glb1l3* in situ hybridization and Brn3b immunohistochemistry. *Glb1l3* mRNA was mainly expressed in the rods, in the cells of the INL—including horizontal and bipolar cells, and in the ganglion cells. OS, outer segments; ONL, outer nuclear layer; INL, inner nuclear layer; GCL, ganglion cell layer.

In the absence of a reliable antibody against Glb1l3, we further investigated the localized expression of its transcript in the retinal cell layers by combining in situ hybridization and immunohistochemistry. In situ hybridization was followed by immunohistochemical analysis using cell-type specific retinal markers to label rods (recoverin), cones (cone arrestin), horizontal cells (calbindin), bipolar cells (PKCα), Müller cells (GFAP), and ganglion cells (Brn3b). As the staining for Brn3b was too weak to be detected in a combined experiment, two adjacent retina sections were used for Glb1l3 in situ hybridization and Brn3b immunohistochemistry. As shown in Figure 6, Glb1l3 transcript was mainly expressed in the rods, in the inner retinal cells, including horizontal and bipolar cells, and in the ganglion cells.

### Age-related increased expression of *Glb1l3* in the retina

**Figure 7:**
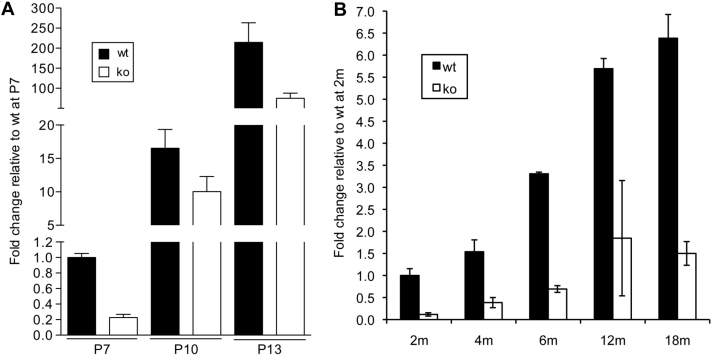
Age-related increased expression of Glb1l3 in the retina. **A**: mRNA expression from wt (black squares) and *Rpe65^−/−^* retinas (white squares) was assessed by real-time PCR in differentiating retinas from post-natal day (P) 7 to P 13 (P 7–13). **B**: Retinal mRNA expression was assessed by microarray in adult retinas from 2 to 18 months of age (2–18 m). In wild-type (WT) mice, Glb1l3 transcript was strongly induced from P 7 to P 13 and age-dependent increased expression still persisted in adulthood and during aging (p<0.0001 by two-way ANOVA). In *Rpe65^−/−^* mice, the level of *Glb1l3* mRNA was reduced in differentiating (p<0.05 by two-way ANOVA) and adult (p<0.0001 by two-way ANOVA) retinas as compared with wt mice. Data are the mean±standard error of the mean (SEM) of 3 independent animals.

We further assessed the expression of *Glb1l3* during postnatal retinal development in WT and *Rpe65*^−/−^ mice from postnatal day (P)7 to P13 (Figure 7A). In WT retinas, *Glb1l3* mRNA was weakly detectable at P7, as reflected by the high cycle threshold (C_t_) value of 34 measured in real-time PCR amplification (data not shown). However, its expression was strongly induced in the differentiating retinas from P7 to P13, by over 16-fold at P10, and by over 200 fold at P13 (p<0.0001). In addition, the age-related increase in *Glb1l3* expression persisted, although at a slower rate, in adulthood and during aging up to 18 months of age. An overall sixfold induction of *Glb1l3* mRNA was observed from 2 to 18 months of age (p<0.0001; Figure 7B). In *Rpe65*^−/−^ retinas, although *Glb1l3* mRNA also increased during retinal differentiation and aging, its level of expression was reduced by two- to fourfold, as compared with WT retinas (p<0.0001).

## Discussion

In the present study, we report the retinal expression of the β-galactosidase-related members belonging to the glycosyl hydrolase 35 protein family, whose function in retinal tissues has not yet been addressed.

We observed that the expression of *Glb1l3* was reduced in *Rpe65*^−/−^ mice before the onset and during the course of the disease. Altered expression started in the differentiating retina and persisted during aging, indicating that the early downregulation of *Glb1l3* is mostly due to the absence of 11-*cis* retinal rather than to the progression of retinal degeneration. *Glb1l3* was expressed in the retinal layers and the RPE/choroid. In comparison, the other Glb-related members were ubiquitously expressed in the different ocular comparents and mostly remained unchanged during progression of the disease.

We also found a strong induction of *Glb1l3* expression in the healthy retina during postnatal retinal development. This coincides with the terminal differentiation of the photoreceptors at a time when components of the phototransduction become functional. At a lower rate, the increase in Glb1l3 persisted during adulthood and aging, supporting a physiologic role of Glb1l3 in the retina to confront age-dependent metabolic stress. Altogether, these results indicate that Glb1l3 may play an important role in retinal cell homeostasis, which may be perturbed in the absence of the visual chromophore.

It has been established that the production of lactate is the usual route of glucose metabolism in retinal cells [15]. The electric activity of the retina depends on available glucose from retinal vessels. In the rod outer segments, high amounts of glucose are necessary to restore, through the pentose phosphate pathway, nicotinamide-adenine dinucleotide phosphate (NADPH) used by the retinol dehydrogenase to reduce all-*trans* retinal to all-*trans* retinol following the phototransduction cascade [16]. We speculate that Glb1l3 may contribute to the production of lactate in retinal cells through the cleavage of lactose provided from the blood into glucose and galactose. A reduced level of Glb1l3 may affect lactate availability and the metabolic functions of the photoreceptors.

Another putative role of *Glb1l3* may be related to the lysosomal function in retinal neurons. The lysosomal hydrolase Glb1 cleaves the β-linked terminal galactosyl residues from gangliosides, glycoproteins, and glycosaminglycans. Mutations in *Glb1* are the cause of two human diseases, GM1 gangliosidosis and Morquio disease type B [17]. The latter is a rare autosomal recessive disorder characterized by the accumulation of keratan sulfates in the cornea, skeletal tissues, and cartilage, causing massive spondyloepiphyseal dysplasia. GM1 gangliosidosis, an autosomal recessive lysosomal disorder, is characterized by the accumulation of GM1 and GA1 gangliosides in nerve cells and by the increase in glycosaminoglycans and glycopeptides in visceral organs and other tissues [18,19]. Pathological abnormalities have also been found in the retinas of GM1 patients, involving membranous cytoplasmic bodies, ganglion cell loss, and optic nerve atrophy [20]. Excessive accumulation of gangliosides leads to visual loss in affected patients [21,22]. Moreover, the gangliosidosis mouse model deficient in GM1 β-galactosidase displays neuropathological defects similar to those identified in the retinas of human patients [23].

In summary, deficiency of the *Rpe65* isomerase gene has many consequences, some of which may be related to impaired metabolic functions in the retinal neurons. Aberrant RPE cells may thus affect the metabolism of the underlying neuroretina.
